# “The Red Spots Are Now Lava, We Shouldn’t Step on Them”—The Joint Creation of Novel Arbitrary Social Contexts in Pretend Play

**DOI:** 10.1162/opmi_a_00082

**Published:** 2023-06-09

**Authors:** Krisztina Andrási, Ildikó Király

**Affiliations:** Doctoral School of Psychology, ELTE Eötvös Loránd University, Budapest, Hungary; Institute of Psychology, ELTE Eötvös Loránd University, Budapest, Hungary; MTA-ELTE ‘Lendület’ (Momentum) Social Minds Research Group, Institute of Psychology, ELTE Eötvös Loránd University, Budapest, Hungary

**Keywords:** pretend play, social cognition, social conventions

## Abstract

Pretend play has been extensively studied in developmental science, nevertheless important questions remain about how children engage in and navigate between pretend episodes. In this proposal, we scrutinize childhood pretense from a social cognitive developmental point of view. First, we review previous theories of pretend play structured around important questions that pinpoint some attributes of pretend episodes, such as their transient and socially defined nature. In these sections, evidence is also reviewed about children’s understanding of these attributes. Following this, we describe a novel proposal of pretend play which extends recent accounts of (pretend) play (Wyman & Rakoczy, 2011; Chu & Schulz, 2020a) by exploiting the importance of social interactions in pretense. We contend that engaging in shared pretending can be considered a manifestation of and support for children’s ability to participate in and set up arbitrary contextual boundaries with others. These claims are discussed with regards to how pretend play may figure into social development, its potential implications for intra- as well as intercultural variation, as well as future research.

## INTRODUCTION

“On this rug, the red spots are now lava, we shouldn’t step on them”—was declared spontaneously by a preschooler in our lab. From early on, these kinds of activities are ubiquitous in the everyday life of children. Starting from their second year of life, while they are still actively learning about the world, children seem to be capable of construing the world as it is not (Lillard et al., 2011). A number of concepts have been introduced to describe this form of activity, such as make-believe, symbolic or pretend play (Harris et al., 1993; Piaget, 1952; Walton, 1990). One crucial attribute of pretending is that it is guided by some form of mental representation that results in nonliteral behaviors or actions (Fein, 1981; Lillard, 2001; Weisberg, 2015). Children start to engage in simple pretend scenarios from around 18-months of age (or earlier, see: Fenson & Ramsay, 1981; Tamis-LeMonda & Bornstein, 1994), while they also adequately recognize pretense and share pretend scenarios with others (Haight & Miller, 1992, 1993; Onishi et al., 2007). Remarkably, pretense seems to be universal—it even appears in cultures where children are not encouraged to play (Gaskins et al., 2007; Smith, 2005). Despite being one of the trademark characteristics of human childhood, the cognitive background and function of pretend play still raises open questions.

In the following sections, first, we review previous theoretical accounts of and empirical findings about pretend play, organized around some important questions with regards to its developmental role and interactional roots, as well as pertaining to children’s understanding of certain characteristics of social pretend episodes. Following this, we suggest extensions for previous proposals of social pretend play through drawing attention to the arbitrary and provisional social contexts created during pretense. We propose that engaging in pretense manifests and supports children’s ability to set up transient, arbitrary contexts with others (Chu & Schulz, 2020a; Rakoczy, 2007, 2008b). In the last sections, the details and implications of this proposal are further discussed.

## WHAT KIND OF ROLE ENGAGING IN PRETEND PLAY MAY PLAY IN COGNITIVE DEVELOPMENT?

Even though people intuitively endow pretend play with benefits for children, its role in cognitive development remains a question. Some approaches suggest that early pretending appears as a consequence of some newly emerging cognitive process, such as the ability to represent the world (Piaget, 1952) or to handle multiple models about reality (Perner, 1991; these approaches jointly described as “*pretense as process*” theories by Friedman & Leslie, 2007). In this sense, pretending may be the (behavioral) manifestation of a certain ability or process, but would not necessarily contribute to its development.

Alternative theories posit that engaging in pretense serves an important role in cognitive development. For example, it has been assumed to contribute to the development of counterfactual reasoning (Harris, 2000; Walker & Gopnik, 2013; Weisberg, 2015), creativity (Carruthers, 2002; Nielsen, 2012) or executive functions (Carlson & White, 2013; Thibodeau-Nielsen et al., 2020). Further proposals highlight its importance in reasoning about social others—through figuring into the development of children’s theory of mind abilities (Leslie, 1987, 1994), or their understanding of social realities (Rakoczy, 2007). Others have argued that pretending supports children in acquiring culture specific skills, knowledge and institutional practices (Adair & Carruthers, 2023; Wyman, 2014) and that it enables children to develop their executive function skills in culture-specific ways (Doebel & Lillard, 2021).

The exact contribution of pretend play to cognitive development is difficult to investigate. The abilities that are studied in connection with pretense may be linked with one another, while creating control groups who do not engage in pretense is almost impossible and ethically questionable, therefore making the causal links difficult to disentangle (Weisberg, 2015). Nevertheless, a recent review examined empirical evidence to explore the connection of pretense with other abilities (Lillard et al., 2013). The empirical data is weak and spare with respect to direct causality; pretense might have a crucial role in the development of language, narrative abilities and emotion regulation or these abilities might enable, or merely be correlated with pretense. Correlations, but little evidence for causation, also applies to executive function abilities, social skills and reasoning. When it comes to theory of mind abilities and creativity, empirical findings are inconsistent, and may lend support to the view that a third variable is causing the association. All in all, pretend play is associated with the development of a number of abilities, but the exact way in which it may figure into their advancement appears to be complex and variable.

## THE ROOTS OF PRETEND PLAY: INDIVIDUAL OR SOCIAL?

Closely intertwined with the previous question is whether pretending is mainly rooted in intra- or inter-psychological processes (Nielsen & Christie, 2008). Approaches to pretending tend to either focus on the individual (pretend production and the intra-individual processes), or on the importance of social partners in its emergence. In the former view, pretending appears in childhood as a consequence of some newly emerging cognitive process (see Piaget, 1952; Perner, 1991). Accordingly, while infants and children may engage in pretense with others, this is rooted in intra-individual cognitive processes that develop at certain ages. In contrast, alternative theories argue that pretend play is fundamentally social and communicative starting from early development (Friedman, 2013; Friedman & Leslie, 2007; Leslie, 1987), that it is first produced interpsychologically (Adair & Carruthers, 2023; Nielsen & Christie, 2008; Rakoczy, 2007), and highlight the importance of adult scaffolding in its development (Rakoczy et al., 2005; Vygotsky, 1967). Relatedly, according to Rakoczy and colleagues (Rakoczy, 2007, 2008b), pretending with others can be considered early evidence for shared intentionality—in case of which, two or more persons share an intentional “we” attitude that cannot be reduced to their individual intentions—which is ubiquitous in adulthood.

The question whether early pretend acts are rooted in social interactions or not is difficult to investigate. On the one hand, observational data could shed light on the amount of pretending children engage in with others and alone, as well as how the proportion of this may change with development. However, since children take part in social interactions from birth, even if they play individually, this still could be built on their interactive experiences with others—and vice versa. Evidence shows that early pretense is highly scaffolded and initiated by parents and older siblings (Dunn, 1988; Haight & Miller, 1993). Additionally, findings demonstrate that social pretend episodes are accompanied by communicative signals, such as eye contact, from age one, and during their second year of life, children engage in pretense through replicating others’ pretend actions (Howes et al., 1989). Also, from around 18 and 24 months of age, children adequately interpret and respond to simple pretend acts by others (Dunn & Dale, 1984; Haight & Miller, 1993), and make appropriate inferences about their behavior (Harris et al., 1993).

Another line of research explored whether children’s pretend acts tend to be imitative or creative (Nielsen & Christie, 2008; Rakoczy et al., 2005; Striano et al., 2001). The results suggest that the pretend acts of preschoolers are characterized by imitation and are heavily accompanied by communication such as frequent gaze-alteration and smiling (Rakoczy et al., 2005; Striano et al., 2001). At the same time, the proportion of creatively invented pretend acts increases with age and adult modeling results in the production of novel ideas and more complex playing (Nielsen & Christie, 2008; Striano et al., 2001). These results suggest that elaborate pretend episodes with creatively produced elements in preschoolers—that could be played individually or in groups—may be rooted in observing others pretend and sharing simple pretend episodes with them. Nevertheless, it is important to note that the complementarity one could intuitively associate with “genuinely” joint pretense only appears as around the age of 3 (Howes et al., 1989). Thus, we are not able to conclusively discern how children understand and participate in these scenarios before the appearance of language—similarly to the case of other interactions, such as joint attentional situations. However, the results suggest that even young children can recognize and engage in pretending with others, and their pretense behavior cannot be disentangled from what they experience in social settings; thus highlights the importance of social context in pretending.

## DO CHILDREN GRASP THE TRANSIENT, SOCIALLY DEFINED NATURE OF PRETEND EPISODES?

As mentioned above, according to Rakoczy and colleagues (2007, 2008b; Searle, 1975, 1995; Walton, 1990; Wyman, 2014), joint pretense is an early instance of shared intentionality: pretending with others can be conceived as a collective activity that includes the assignment of transient status functions and made up constitutive rules (“This banana is now a telephone in our game”). Pretend play, hence, is governed by (pretend) stipulations that exist purely based on the agreement of the participants and which are applicable within the thus created framework.

It is an empirical question to explore whether children grasp these characteristics of shared pretend episodes. In order to appropriately engage in these, children need to follow pretend stipulations and their implications, and track the boundaries of the episodes. This entails quarantining the pretend representations, while at the same time, applying the stipulations at certain times and places, and to particular partners. This latter not only requires separating the representations, but also switching between contexts that implicates the involvement of executive functions.

### Following Pretend Stipulations

Fifteen month-old infants detect pretend violations in others’ actions and behave in accordance with simple pretend scenarios (Bosco et al., 2006; Onishi et al., 2007). From around their second birthday, they draw appropriate inferences about the pretend behavior of their partners in more complex situations (Harris et al., 1993; Ma & Lillard, 2006; Tomasello et al., 1999). Preschoolers are even more proficient: they keep track of the identity of objects in different games (Weisberg & Bloom, 2009; Wyman et al., 2009a). Two-year-olds also expect others to follow current stipulations (Rakoczy, 2008a). Thus, children follow pretend stipulations and their implications for social partners from their second year of life.

### Tracking the Boundaries of Pretend Episodes

Empirical evidence shows that by 3 years of age, children are able to separate reality from pretense (Bourchier & Davis, 2002; Flavell et al., 1987; Woolley & Phelps, 1994), and separate pretend contexts from one another (Weisberg & Bloom, 2009; Wyman et al., 2009a). This is less clear in the case of younger children; nevertheless, even though they spend much time engaged in pretense, this does not result in mistaken beliefs. Thus, these representations seem to be appropriately quarantined from their developing knowledge about the world (Leslie, 1987). Importantly, this boundary is not always strict, for example, children can learn and generalize from pretend episodes (Hopkins et al., 2015; Sutherland & Friedman, 2012, 2013).

#### What cognitive architecture enables tracking the boundaries of pretend episodes?

One of the most influential debates about pretending concerns the representations enabling the quarantining of pretend episodes. According to Leslie and colleagues (Friedman & Leslie, 2007; Leslie, 1987, 1994), pretense is supported by metarepresentations even from young infancy. This format enables the decoupling of the regular relations of the content and thus making it available for pretense via ascribing agent-centered representations to the participants. As a result of this decoupling, the representational system and children’s semantic knowledge remains intact. Stich and colleagues (Nichols & Stich, 2000) also argue that representations involved in pretense can have the same format and content as beliefs, however, they claim that ascribing agent-centered representations is not necessary to participate in pretense. Rather, representations are quarantined in a so-called Possible World Box which is a “(…) work space in which our cognitive system builds and temporarily stores representations of one or another possible world” (Nichols & Stich, 2000, p. 122). Recent evidence indeed suggests that children grasp that mental states are involved in pretending, and thus favors the metarepresentational view (Weisberg, 2015).

### Constraining Pretend Stipulations by Time, Space, and Partners

The contextual boundary within which pretend stipulations are valid may be designated by a number of factors—such as time, space or participants of the episode. While sensitivity to temporal boundaries have not been tested specifically, two year olds follow that the same object may have changing identities in subsequent pretend scenarios (Harris et al., 1993). Preschoolers also understand that different stipulations may apply at different locations (Wyman et al., 2009a) and extend this to others (Wyman et al., 2009b, Experiment 2). Additionally, they follow to whom a stipulation is known and applicable (Andrási et al., 2022; Hickling et al., 1997; Kalish et al., 2000; Wyman et al., 2009b, Experiment 1). This suggests that they understand that stipulations are valid within constraints by time, location and specifically, by partner.

### Context-Switching

Flexibly navigating social contexts not only requires separating the relevant representations, but also executive function skills. Lillard and colleagues (Lillard et al., 2013) suggest a relationship between pretending and executive functions, with pretend play being one of the developmental routes for executive function skills. Recent studies also support that these abilities are related (for e.g., Carlson et al., 2014; Thibodeau-Nielsen et al., 2020). Inhibitory control skills seem to be specifically related to engagement in social pretending (White et al., 2021). Findings from other investigations, however, did not find a straight-forward relationship between these abilities (see Doebel & Lillard, 2021).

## CREATING ARBITRARY SOCIAL CONTEXTS IN PRETEND PLAY

Previous findings suggest that early pretend play is heavily scaffolded by social partners, and that from the preschool years, children are able to competently navigate multiple episodes of pretend play with various partners (Weisberg, 2015). Preschoolers also grasp some important attributes that characterize the social contexts created in shared pretense. While pretend play is associated with the development of a number of cognitive skills—many of which are important for socio-cognitive development (such as language and narrative abilities, emotion regulation, social skills or theory of mind)—the exact manner in which it may figure into their development remains to be explored.

Building on these findings, we propose that pretend play both manifests and supports children’s ability to recognize and navigate the boundaries of social contexts with others, as well as to create novel social contexts. Humans have a fundamental motivation to maximize their learning benefit about their environment and they acquire most of their knowledge from conspecifics (Herrmann et al., 2007). That is why it is so important for them to establish a shared representational space with fellow humans, namely to get and maintain access to their knowledge base (Oláh et al., 2019). In joint pretense, novel social contexts are created that determine the action utilities and beliefs of those involved. These contexts are arbitrary and transient, and are applicable to certain places, times and people. As children participate in pretense with social partners, they gain experience in joining and creating these social contexts, which may be one of the developmental routes for skills related to recognizing, creating and navigating social-contextual boundaries in later life. The fact that pretend scenarios are transient and result in propositions which are true within the pretend context but false outside of it (Cosmides & Tooby, 2000), may prompt children to pay attention to the contextual boundaries. This may support them in identifying *who shares their knowledge*, in creating novel social contexts, and in identifying the boundaries of already existing social communities.

These abilities would be important, among other things, because much information in children’s environments is constrained by being “true” only temporarily or locally. Indeed, it has been proposed that one of the remarkable characteristics of humans is that they can successfully modify their behavior tailored to the specific situation based on contingent information (Cosmides & Tooby, 2000). Additionally, human social interactions are often guided by social conventions which are context-dependent and arbitrary (Rakoczy & Schmidt, 2013; Lewis, 1969). Therefore, the validity of cultural information is generally constrained to places, times and social communities—and thus shares many similarities with social contexts created during pretend play.

As to why children pretend, we suggest that their motivation is built on a general motivation of becoming expert members in social contexts (Király, 2019). This fundamental drive, process fuelled by curiosity, motivates children to share in the knowledge of their partners. Pretend play may offer an opportunity to gain insight both in the formal characteristics of social contexts, as well as specific knowledge through the involvement of culture specific scripts of events in play (Wyman, 2014). In addition, we posit that humans as a species enjoy setting up simple, transient contexts with context-dependent rules, and this makes them better at creating flexible and novel contexts—such as new communities or groups—which are ubiquitous in their social world. However, we would suggest that children’s motivation for social pretense would also be impacted by their cultural environment. In the following sections, we briefly expand on how this proposal ties together ideas from two prominent approaches, as well as its potential implications for development and cultural differences.

### Theoretical Roots

Theoretically, this proposal is closely connected to, on the one hand, a theory of playing recently proposed by Chu and Schulz (2020a). The authors suggest that one of the most important characteristics of the unique form of playing that emerges after toddlerhood is the invention of novel problems. This entails the “players” intervening in regular utility functions which would otherwise frame their behavior, and incurring unnecessary costs and aiming for arbitrary, self-invented rewards. In this view, engaging in play scenarios could be useful for supporting the generation of new problems and goals which may lead to novel thoughts and ideas, and is often motivated by curiosity. Additionally, the authors also address the arbitrary nature of play and propose the following: “(…) the idiosyncratic, arbitrary nature of the problems set in play, and the often flimsy, inadequate solutions generated, may be offset by the fact that the ideas generated in play can be decoupled from the problems that inspired them and be valuable in their own right.” (Chu & Schulz, 2020a; 14.11). In other words, although the problems invented during play may be arbitrary, these can inspire solutions that could be useful in a future context. Importantly, this theory focuses on solitary as well as collaborative play and proposes that, in the sense of inventing arbitrary constraints, many kinds of play involve a kind of “pretending” or “making up” goals (Chu & Schulz, 2020a, p. 14.12).

On the other hand, it is also rooted in the proposal of Rakoczy and colleagues which posits that participating in pretend play supports children’s developing understanding of social realities (Rakoczy, 2007; Wyman, 2014; Wyman & Rakoczy, 2011). In more detail, pretend play shares similarities with adult social institutions—for example, it involves the assignment of status functions –, yet its structure is much simpler. For example, it only applies to a small number of people and does not constitute part of a wide web of function assignments (Wyman & Rakoczy, 2011). This deems participating in these episodes suitable as a cradle for understanding more complex social institutions (Rakoczy, 2007) and allows children to have a rudimentary grasp on the fact that fictional status can be assigned by joint intention (Wyman, 2014).

We believe our proposal ties together and expands on these approaches through integrating important insights about pretend episodes. First, the theoretical approach of Rakoczy and colleagues highlights that creating pretend stipulations with others resembles the operation of social institutions in multiple aspects. We enrich this idea—arguing that as pretending supports children in creating, recognizing and navigating the boundaries of social contexts, this may figure into the development of a number of social skills necessary for navigating multiple social communities in adulthood, within which the capacity to re-identify members for a given set of shared knowledge is essential, since it could guide the organization of knowledge base. Second, as Chu and Schulz (2020a) observe, pretend episodes are characterized by modified utility functions with arbitrary rewards. We broaden this argument for social pretense, by emphasizing that utility functions and their rewards are rooted in the pretend stipulations created by the group and thus determine the behavior of both the child and the other participants. Even though problems tackled in play may be arbitrary, these could inspire solutions in future contexts. This consequence has social aspects as well: ideas decoupled from their original contexts might be shared with others. Furthermore, if pretending figures into abilities related to creating social groups, this contributes to the creation of novel social contexts which, in turn, may increase the probability of the ideas itself becoming useful in future contexts. Arbitrariness itself is a characteristic of social conventions that regulate everyday life (Lewis, 1969; Rakoczy & Schmidt, 2013). Thus, having experience with social pretense may also support children in grasping that human social interactions are often regulated by agreed upon, arbitrary utility functions.

### Empirical Implications and Questions

We propose that pretending is one of the developmental routes for abilities connected to recognizing, creating and navigating social-contextual boundaries, but not an exclusive one. Another potential route is through gaining experience with how word meanings may be shared among members of wider or smaller communities. Relevant evidence shows that toddlers expect common names to be known by different individuals (Graham et al., 2006; Henderson & Graham, 2005), but older children do not expect those without specific experience to be familiar with proper names (Birch & Bloom, 2002; Diesendruck, 2005, Experiment 1) or people to know common names from another language than their own (Diesendruck, 2005, Experiment 2). Another route could be to gain experience with the conventional use of objects and how these may be known and shared by members of cultural communities. Evidence suggests that by the age of two, children understand some aspects of the conventionality of objects: two year olds expect others to use the same object for the same function (Casler & Kelemen, 2005), but their expectations are also guided by cues of shared cultural knowledge (Oláh et al., 2014; Pető et al., 2021). These findings show that children are sensitive to conventionality, thus experience with language and objects could serve as potential routes for understanding contextual boundaries.

Relatedly, as there are multiple developmental routes for these abilities, individual differences in pretending would not necessarily result in differences in adulthood. However, we would predict that relevant socio-cognitive abilities—such as tracking other people’s knowledge states or their changing utility functions, as well as the boundaries of social contexts—are facilitated in pretend play scenarios. At the same time, we would expect to find cultural differences, with pretend play being more diverse, frequent and pervasive during childhood in societies where children are members of and encounter more communities and institutions which would require them to represent contextually bound information.

#### Development and individual differences.

Empirical findings reflect that from early on, children are proficient in navigating the boundaries of social pretend episodes (Harris et al., 1993; Weisberg & Bloom, 2009; Woolley & Phelps, 1994; Wyman et al., 2009a). As mentioned above, we would predict relevant skills to be facilitated in pretend scenarios. Findings suggests that this is true in the case of theory of mind abilities—children report their own past belief and the false belief of others accurately in case they need to report a previous pretend stipulation at earlier ages than it is the case with “real” state-of-affairs (Andrási et al., 2022; Gopnik & Slaughter, 1991; Hickling et al., 1997; Kalish et al., 2000). We would predict this to be true in other abilities as well, for example, for how the utilities of another person change from context to context.

Relatedly, it is an open question how children reason about the way action utilities may vary between pretend episodes or between social contexts for the same person. To our knowledge, no study has specifically addressed this question. On the one hand, as expanded on above, children can adequately follow changing pretend stipulations and their implications for themselves and for others. This would suggest that they could also monitor changes in utilities. Recently, it has been shown that both toddlers and children reason about other people’s behavior based on the assumption that others choose actions to maximize utilities—and make adequate inferences about other people’s costs and rewards based on their choices (Jara-Ettinger et al., 2015, 2017). Relatedly, they grasp that the same action may have different costs for different people (Jara-Ettinger et al., 2015) and can apply their reasoning about the utilities of others when deciding what to teach them (Bridgers et al., 2020), but it remains a question whether they grasp that the same action may have different costs for the same person. Based on our account, we would predict that it would be easier for children to follow how utilities may change for another individual in pretend scenarios (at an earlier age and in more complex situations). At the same time, it would be interesting to explore whether children’s inferences about the utilities of their partners differs in pretend play. For example, a seemingly most costly manner of doing something may be the reward itself while pretending (see Chu & Schulz, 2020b), therefore otherwise warranted inferences about competence or preference need to be reconsidered.

#### Differences between cultures.

The presence of pretend play seems to be universal in all cultures (Gaskins, 2013; Smith, 2005). At the same time, the frequency, content, and developmental trajectory of pretending, as well as parental attitudes towards it vary between cultures (Gaskins et al., 2007; Haight & Miller, 1993; Haight et al., 1999). A number of interpretations have been proposed to explain these differences, including children engaging less in pretend play in cultures where they get more opportunities to practice culturally relevant skills via other activities and children who live in words which are less complex and open-ended (Gaskins et al., 2007). We believe our account can be an interesting addition to this question through highlighting that children could be more inclined to engage in social pretense—both more frequently and more persistently in childhood –, and the thus created social contexts, in societies where they encounter more communities and social institutions. This remains an interesting empirical question.

## CONCLUSIONS

The social aspects of pretend play are important to examine as, from early on, children recognize pretense in others and engage in shared pretending with them. These shared play scenarios result in an agreed upon set of representations that guide the behavior of game partners—which, in many ways, resemble the socially constructed, seemingly unwarranted shared beliefs that determine social interactions in the life of adults on a much bigger scale (Cosmides & Tooby, 2000). We propose that engaging in social pretense supports children in recognizing, creating and navigating the boundaries of social contexts. While pretending with others, children participate in and set up arbitrary contexts with others and they need to apply representations created in pretense in a context dependent manner. Being sensitive to the boundaries of these contexts is crucial, on the one hand, as children are members of a number of smaller or wider communities with varying expected shared knowledge and norms, and on the other hand, in order to navigate successfully in societies that are becoming increasingly multicultural.

## AUTHOR CONTRIBUTIONS

Krisztina Andrási: Conceptualization, Writing – Original draft. Ildikó Király: Conceptualization, Funding acquisition, Supervision, Writing – Review and editing.

## ACKNOWLEDGMENTS

The authors would like to thank Réka Petö and the anonymous reviewers for their valuable comments on the manuscript.

## FUNDING INFORMATION

The writing of this Perspectives article was supported by the MTA Momentum program (LP-2017-17/2017, PI: Ildikó Király).

## DATA AVAILABILITY STATEMENTS

No data was collected in connection with this article.
